# Multi-Compartmental Dissolution Method, an Efficient Tool for the Development of Enhanced Bioavailability Formulations Containing Poorly Soluble Acidic Drugs

**DOI:** 10.3390/pharmaceutics15030753

**Published:** 2023-02-24

**Authors:** Miklós Tamás Katona, Lili Nagy-Katona, Réka Szabó, Enikő Borbás, Péter Tonka-Nagy, Krisztina Takács-Novák

**Affiliations:** 1 Department of Pharmaceutical Chemistry, Semmelweis University, 7 Hőgyes Endre Street, H-1092 Budapest, Hungary; 2 Egis Pharmaceuticals PLC, 116-120 Bökényföldi Street, H-1165 Budapest, Hungary; 3 Department of Organic Chemistry and Technology, Budapest University of Technology and Economics, 3 Műegyetem rakpart, H-1111 Budapest, Hungary

**Keywords:** multi-compartmental dissolution, solubility, supersaturation, IVIVC, ibuprofen, BCS Class IIa

## Abstract

The purpose of this study was to investigate the applicability of the Gastrointestinal Simulator (GIS), a multi-compartmental dissolution model, to predict the in vivo performance of Biopharmaceutics Classification System (BCS) Class IIa compounds. As the bioavailability enhancement of poorly soluble drugs requires a thorough understanding of the desired formulation, the appropriate in vitro modelling of the absorption mechanism is essential. Four immediate release ibuprofen 200 mg formulations were tested in the GIS using fasted biorelevant media. In addition to the free acid form, ibuprofen was present as sodium and lysine salts in tablets and as a solution in soft-gelatin capsules. In the case of rapid-dissolving formulations, the dissolution results indicated supersaturation in the gastric compartment, which affected the resulting concentrations in the duodenum and the jejunum as well. In addition, a Level A in vitro–in vivo correlation (IVIVC) model was established using published in vivo data, and then the plasma concentration profiles of each formulation were simulated. The predicted pharmacokinetic parameters were consistent with the statistical output of the published clinical study. In conclusion, the GIS method was found to be superior compared to the traditional USP method. In the future, the method can be useful for formulation technologists to find the optimal technique to enhance the bioavailability of poorly soluble acidic drugs.

## 1. Introduction

The formulation of poorly soluble drug substances into dosage forms with proper pharmacokinetics is a challenge for both original drug discovery and generic/value-added generic drug development. As the number of drug candidates is shifting towards high lipophilicity and poor water solubility, the importance of formulation strategies to enhance bioavailability is increasing [1]. In the case of BCS Class II (low solubility and high permeability) drugs, improved absorption can be achieved by increasing the dissolution rate of the formulation [2,3]. For this purpose, salt formation with the drug substance is perhaps the most common approach; however, pre-dissolving the drug in a lipid-based formulation, applying amorphous structures, or reducing particle size are also well-known techniques [4,5,6].

The in vitro dissolution testing is an important tool for characterizing the biopharmaceutical properties of a drug product at different stages throughout its life cycle. Compliance with the dissolution requirements ensures that the finished drug product is consistent with the release rates of the active pharmaceutical ingredient (API) as determined in bioavailability studies during the clinical trials [7]. The results obtained need to be independent of the testing laboratory, therefore, reproducible methods in standardized equipment are to be used. Immediate-release drug products are generally tested in apparatus I and apparatus II, specified by the United States Pharmacopoeia (USP) [8]. However, such methods are usually not sufficient to represent the complex physiology of the gastrointestinal system. If the aim is to support pharmaceutical development by understanding the in vivo effect of different formulation techniques, the usage of advanced in vitro biopharmaceutics models may be necessary.

In order to achieve better predictivity, pharmaceutical scientists made great efforts to develop dynamic multi-compartmental dissolution systems [9]. A two-compartmental artificial stomach duodenal model (ASD) was published by Vatier et al. for the evaluation of the effect of antacids [10]. The ASD has also been used to aid formulation development and crystal form selection [11,12]. To date, several more complex systems have been reported in the literature, such as TNO gastro-Intestinal Model (TIM) [13], Dynamic Gastric Model (DGM) [14], and Human Gastric Simulator (HGM) [15]. Based on the ASD system, Takeuchi et al. developed the Gastrointestinal Simulator (GIS), which is a three-compartmental model consisting of a gastric, a duodenal, and a jejunal chamber connected by peristaltic pumps. The transfer rates (representing the gastric-emptying rate) were determined using propranolol and metoprolol model compounds by comparing the dissolution results with clinical data [16]. The GIS was successfully applied in several studies to predict the in vivo performance of drugs, investigate the supersaturation phenomena, or evaluate the possible drug-drug interaction with acid-reducing agents [17,18,19,20,21]. In order to achieve better in vitro–in vivo correlation (IVIVC), some of the models are combined with in silico simulations. The published studies focused primarily on BCS Class IIb (and BCS Class IIc) compounds. Despite the promising results achieved with the GIS system, to the best of our knowledge, poorly soluble acidic drugs (BCS Class IIa) have not yet been studied.

Non-Steroidal Anti-Inflammatory Drugs (NSAIDs) are well-known for their anti-inflammatory activities and analgesic, antipyretic effects [22]. In the case of analgesic indication, the patient’s interest is to achieve the onset of pain relief as fast as possible [23], which is reported to be in direct correlation with the serum concentration of the active substance [24]. In general, the majority of NSAIDs have an acidic moiety (carboxylic acid, enol), with p*K*_a_ in the 3–5 range, attached to a planar, aromatic/heteroaromatic functionality [25]. The most widely used NSAIDs, such as diclofenac, ibuprofen, and naproxen, are classified as BCS Class IIa drugs [26,27,28]. These compounds are typically poorly soluble in the acidic gastric media, where the molecules are mostly present in an unionized free acid form. However, due to the ionization, they dissolve at the higher pH of the small intestinal fluids, which, together with high permeability, results in complete or almost complete absorption [26]. Ibuprofen is one of the most common analgesic/antipyretic agents. It is available in over-the-counter (OTC) strengths (100 mg and 200 mg) and prescription strengths (400 mg, 600 mg and 800 mg) as well. In case of OTC dosing, adults and children over 12 years old are advised to take 1 to 2 tablets (i.e., 200 mg to 400 mg) by mouth every 4 to 6 h while symptoms last; the maximum daily dose should not exceed six tablets (1200 mg) in 24 h [29]. In addition to the conventional tablet form, ibuprofen is marketed as different rapid-dissolving formulations (e.g., soft-gelatin capsules and tablets containing sodium or lysinate salts of the API). The rapid onset of the analgesic effect as well as the higher absorption rate of rapid-dissolving formulations, is discussed by several in vivo studies [24,30,31]. Legg et al. published the results of a five-period, crossover pharmacokinetic study in which fasted subjects received five different 400 mg ibuprofen dose equivalent formulations (as 2 × 200 mg tablets/capsules). According to the statistical analysis, ibuprofen-sodium and ibuprofen-lysinate, as well as Advil soft-gelatin capsules, were absorbed significantly faster but to a similar extent to standard ibuprofen formulations [23]. In a recent study, Cámara-Martinez et al. tested two different formulations containing ibuprofen in USP II dissolution apparatus. The tests were carried out using different phosphate and maleate buffers with and without acidic pre-treatment of the tablets. Based on the results, they found the acidic pre-treatment to be important to find proper correlation with in vivo results [32].

The aim of this study was to investigate the applicability of multi-compartmental dissolution methods to predict the in vivo performance of BCS Class IIa compounds. For this purpose, different conventional and rapid-dissolving ibuprofen 200 mg formulations were tested using the GIS system with biorelevant dissolution media. The formulations were also dissolved with the quality control method of ibuprofen tablets according to USP [33], and the predictivity of each method was evaluated. To better understand the dissolution results, the pH-dependent equilibrium solubility of the API in Britton-Robinson (BR) buffers and the equilibrium solubility in biorelevant media were also determined using the saturation shake-flask method. Moreover, a Level A IVIVC model was established based on the GIS dissolution profiles and the clinical data published by Legg et al. [23].

## 2. Materials and Methods

### 2.1. Materials

Four commercially available immediate release ibuprofen-containing products were investigated. The formulations were purchased from pharmacies in the United States and Hungary. The tested products and their active ingredients are listed in Table 1.

Ibuprofen drug substance was purchased from Sigma–Aldrich (Burlington, VT, USA). All chemicals used were of analytical grade. The following chemicals were used: sodium-hydroxide; sodium-chloride; sodium dihydrogen phosphate monohydrate; hydrochloric acid; (Molar Chemicals Kft., Budapest, Hungary); acetonitrile (PanReac AppliChem, Darmstadt, Germany); phosphoric acid; (Emsure ACS. Reag. Ph. Eur., Budapest, Hungary); SIF powder (Biorelevant^TM^, London, UK); and Pepsin (Sigma–Aldrich, Burlington, VT, USA).

### 2.2. Equilibrium Solubility Measurements

The saturation shake–flask method was used to determine the equilibrium solubility of the API [34,35]. The measurements were carried out at 37 ± 0.1 °C. Ibuprofen was added in an excess amount (100–600 mg) to 3–5 mL solvent in a stoppered flask. In case it was necessary, the pH was adjusted to the initial value with 1M NaOH solution after 1 h. The flasks were then placed in a GFL 1092 type shaking water bath (GFL GmbH, Burgwedel, Germany) and shaken at 150 rpm for 6 h. The agitation phase was followed by 18 h sedimentation at controlled temperature. A total of 3 aliquot (10–100 µL) samples were then taken from the saturated solution and diluted to the required extent (2–250x) with the tested medium. The concentration was measured by UV spectroscopy at λ_max_: 264 nm. The UV detection was chosen because of its simplicity, taking into account the literature recommendations [35]. In each medium, the solubility experiments were performed in triplicate. The pH dependence of solubility was determined in BR buffers (a mixture of 0.04 M boric acid, 0.04 M phosphoric acid, and 0.04 M acetic acid titrated to the desired pH with 0.2 M sodium-hydroxide) in the pH range 2–8 (pH = 2.0; 4.0; 6.0; 7.0; 8.0) and in 1 M NaOH (pH = 14). The equilibrium solubility was also investigated in biorelevant media modelling gastric and small intestinal fluid in a fasted and fed state with and without solubilizing agents (pepsin or lecithin and bile acid salts). The tested biorelevant media were Blank FaSSGF, FaSSGF, Blank FaSSIF, FaSSIF, FeSSGF-acetate (without milk), Blank FeSSIF, and FeSSIF. The solutions were prepared according to the media preparation tool of biorelevant.com [36].

### 2.3. Dissolution Testing

The dissolution tests were carried out using an Agilent 708 DS dissolution apparatus (Agilent Technologies, Inc., Santa Clara, CA, USA). The media were thermostated at 37 ± 0.5 °C. Each formulation in each method was tested on six parallel samples.

#### 2.3.1. USP Dissolution Method

The tests were performed in a USP II (paddle) apparatus. The dissolution medium was pH 7.2 ± 0.5 phosphate buffer solution prepared by dissolving 6.89 g NaH_2_PO_4_.H_2_O in 1 L distilled water, and the pH was adjusted with 3 M NaOH solution. The samples were placed into a 900 mL medium and stirred at 50 rpm. Samples at 5, 15, 30, 45, and 60 min were taken into HPLC vials via autosampling. The volume of each sample was ~1.2 mL. The sampling cannulas were equipped with 10 µm PVDF full-flow filter tips (Agilent Technologies, Inc., Santa Clara, CA, USA).

#### 2.3.2. GIS Dissolution Method

The GIS was implemented in a dissolution apparatus equipped with 250 mL small-volume vessels, according to Chinese Pharmacopoeia [37]. The system consisted of three main compartments (250 mL vessels) modelling the stomach, duodenum, and jejunum. The vessels were connected to each other by Gilson Minipuls 3-type peristaltic pumps (Gilson Inc., Middleton, WI, USA). At the beginning of the test, 50 mL pH1.6 gastric fluid and 250 mL water were poured into the stomach, 50 mL pH 6.5 intestinal fluid into the duodenum, and the jejunal chamber was left empty. Two additional vessels were used to model the inner fluid secretion into the stomach (pH 1.6 gastric fluid) and the duodenum (pH 6.5 intestinal fluid concentrate), both the stomach and duodenum had a flow rate of 1 mL/min. The biorelevant dissolution media were prepared, and the tests were conducted with and without biomolecules (pepsin, SIF powder). The composition of the applied buffer solutions is summarized in Table 2.

**Figure 1:**
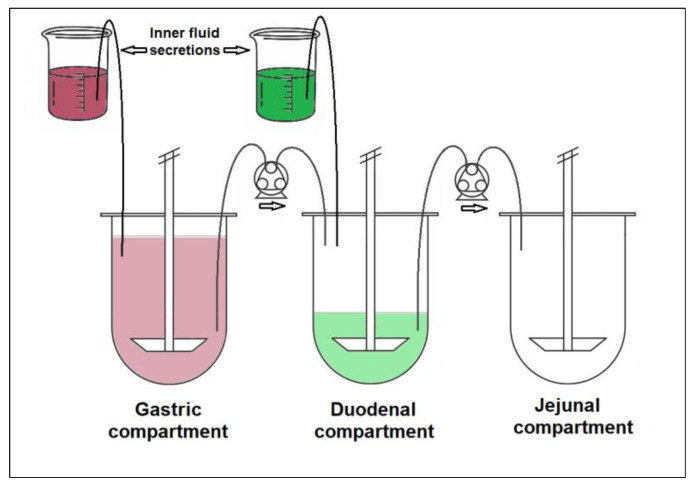
Gastrointestinal Simulator [16].

The tested formulation was dropped into the gastric chamber, and the media were stirred at 50 rpm using rotating paddles. The applied flow rates were 5.5 mL/min from the gastric to the duodenal and 6.5 mL/min from the duodenal to the jejunal chamber, as suggested by Takeuchi et al. [16]. Samples from the compartments were taken manually every 5 min during the 45-min duration of the tests. The duration was limited by the initial fluid volume and the gastric emptying rate. Each sample was filtered through Acrodisc^®^ syringe filters (d = 13mm) with 0.45 µm GHP membrane (Pall Co., Port Washington, NY, USA). The volume of each sample was ~0.5 mL. The schematic diagram of the GIS system is shown in Figure 1.

### 2.4. Determination of Dissolved Drug Content by High-Performance Liquid Chromatography (HPLC)

Waters Acquity-type UPLC device (Waters, Milford, MA, USA) was used to determine the amount of dissolved drug in the solutions. For this purpose, Waters Acquity BEH C18 1.7 µm (2.1 × 50 mm)-type UPLC column was used. The mobile phase was acetonitrile:H_2_O:cc.H_3_PO_4_ = 450:550:1, and the flow rate was 0.7 mL/min. The mode of separation was isocratic. External calibration by five consecutive injections of a standard solution containing the concentration of API corresponding to the approximated concentration of 100% dissolution was applied. The calibration was controlled by the injection of the control standard solution containing the same nominal concentration, followed by the injection of the sample solutions. The absorbance was detected at 214 nm. For standard preparations, accurate measurements were achieved using a Mettler Toledo XP 26 microanalytical balance (Mettler Toledo, Columbus, OH, USA). The sample concentrations in mg/L were calculated using the dilution factor of the standard and the sample solutions and the peak areas of the sample solutions. The chromatographic conditions for each test preparation were the same as the column used for the measurement.

### 2.5. In Vitro In Vivo Correlation (IVIVC)

A Level A IVIVC model was developed using the IVIVC Toolkit 8.3. of Phoenix WinNonlin 8.3.4.295 for Windows (Certara, St. Louis, MO, USA). In vivo data were obtained by digitizing the mean plasma concentration profiles of four different ibuprofen formulations from a fasted state crossover pharmacokinetic study published by Legg et al. [23]. The administered formulations were identical to that of Table 1, except for IBU-Lys. In the case of IBU-Lys, Nurofen Express 342 mg caplets (Reckitt Benckiser, Slough, Berkshire, UK) were administered, the manufacturer of which differed from the formulation used in the in vitro studies. However, the salt form of the active ingredient was the same. A two-compartmental pk model (model 14 of pK tab) was then fitted to the data of each formulation. The gained parameters of Advil 200 mg tablets were implemented to the unit impulse response (UIR) function, and the fitted plasma concentration curve was deconvolved, resulting in the calculated fraction absorbed profile. The cumulative dissolution data of the duodenal and jejunal compartments of the GIS measured in blank biorelevant media were fitted with the Weibull equation. The in vitro fraction dissolved and in vivo fraction absorbed of Advil 200 mg tablets were correlated using the Levy plot. Based on the calculated correlation and UIR function, the plasma concentration profile of each formulation was simulated from the fitted dissolution profiles. Finally, the plasma concentrations predicted from the model and the observed data were compared.

## 3. Results and Discussion

### 3.1. Thermodynamic Equilibrium Solubility Measurements

#### 3.1.1. pH-Dependent Solubility

**Figure 2:**
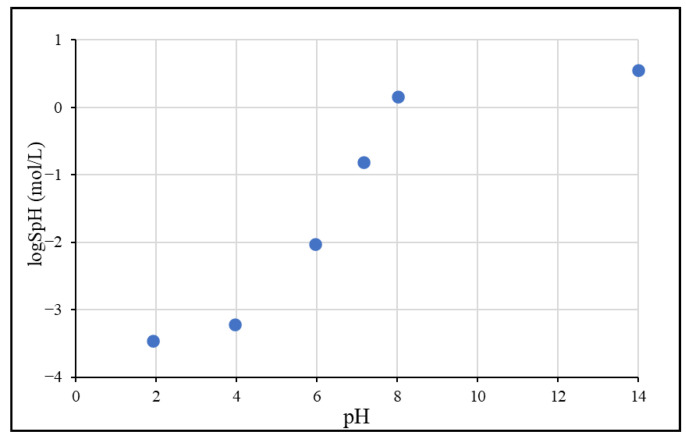
The solubility-pH profile of ibuprofen in Britton–Robinson buffers.

The pH-dependent solubility (S_pH_) of ibuprofen was tested at 5 different pH values in the pH 2–8 range using BR buffer solutions. Additionally, the solubility of the fully ionized form was determined in 1M NaOH solution. The results obtained are summarized in Table 3, and the solubility/pH profile is shown in Figure 2.

As expected from a weak acid compound with p*K*_a_: 4.45, the solubility of ibuprofen is increasing according to the Henderson–Hasselbalch relationship in the pH 2–8 range and reaches a plateau at a higher pH due to the salt formation [38]. Below pH 5, the solubility is low, therefore, it can be assumed that from the formulations, the API can only partially dissolve in acidic gastric media. The measured equilibrium solubility in pH 2.0 BR buffer (logS = −3.46 mol/L) is consistent with the literature intrinsic solubility data (logS_0_ = −3.62) [39]. The small difference might be explained by the fact that the literature data were measured at 25 °C and that at pH 2.0, the molecules are mostly—but not totally—unionized; thus, a slightly higher value is expected compared to the intrinsic solubility.

#### 3.1.2. Solubility in Biorelevant Media

The equilibrium solubility values in biorelevant media are listed in Table 4.

Solubility data in biorelevant media are in accordance with results measured in BR buffers, showing that the pH significantly affects the solubility of ibuprofen. Changing the pH from 1.6 to 4.5, 5.0, and 6.5 results in a 3.4-fold, 7.4-fold, and 44.6-fold increase in solubility, respectively. Pepsin has no effect on solubility at gastric pH. However, the solubilizing effect of natural surfactants of the small intestine further increases the solubility: FaSSIF/FaSSIF blank 1.3-fold; and FeSSIF/FeSSIF blank 5-fold. In the case of FeSSIF, a greater solubilizing effect was observed, which can be explained by the higher concentration of taurocholate and lecithin.

### 3.2. Dissolution Results Obtained by the USP Method

**Figure 3:**
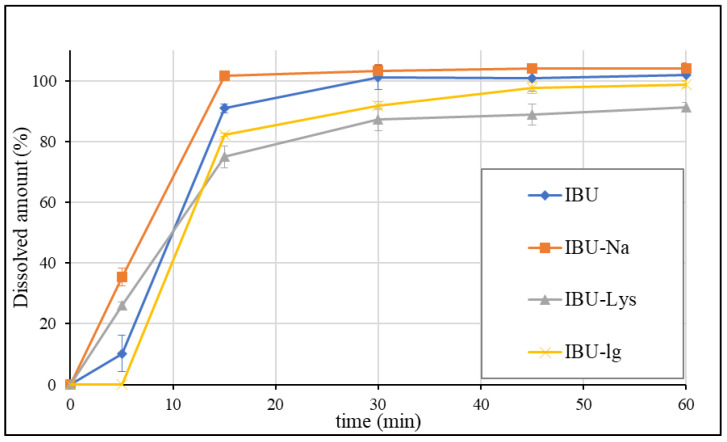
Dissolution results of ibuprofen formulations obtained by USP method.

The USP individual monograph of ibuprofen tablets suggests the dissolution of the formulations at pH 7.2 using USP II (Paddle) apparatus with 50 rpm [33]. The average dissolution profiles of each formulation (1 × 200 mg dosage unit per vessel) are presented in Figure 3.

According to the USP method, IBU and IBU-Na dissolved rapidly, as more than 85% of the drug substance dissolved in 15 min. The mean dissolved amount of IBU-lq was less than 85% in 15 min, however, its dissolution profile can be considered statistically similar to IBU based on the calculated similarity factor (f2 = 55). The dissolution rate of IBU-Lys was found to be significantly slower than that of IBU (f2 = 37). Overall, the USP method was unable to discriminate between rapid-release and standard ibuprofen formulations.

### 3.3. Dissolution Results Obtained by GIS Method

**Figure 4:**
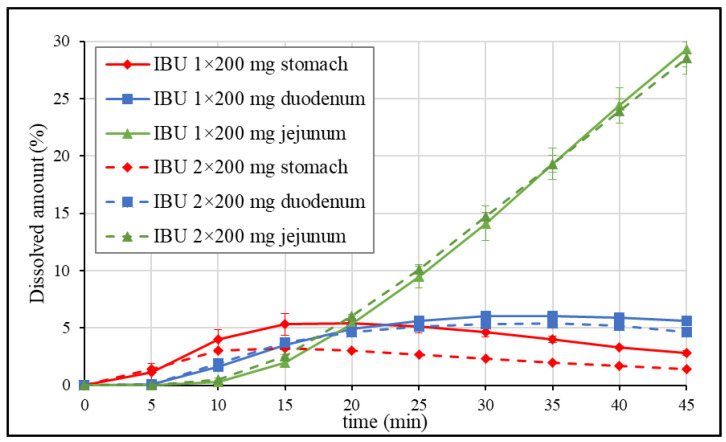
GIS dissolution results of IBU 1 × 200 mg vs. 2 × 200 mg tablets in blank biorelevant media.

Since it is advised to take 1 to 2 tablets of the 200 mg dose strength formulations, 400 mg dose equivalents were administered in the published pharmacokinetic study, and the in vitro dissolution of IBU 1 × 200 mg tablets vs. 2 × 200 mg tablets was compared in blank biorelevant media. The average dissolution profiles in each compartment are presented in Figure 4.

Figure 4 shows that only a small amount of the API was dissolved in the stomach. Both dissolution profiles reached a maximum after 15 min, then a slow decrease was observed. The maximum of the curve represents the time when the gastric emptying rate of the API equals the dissolution rate. The difference in the percentage dissolved in the later stage of the test can be explained by reaching the equilibrium solubility limit (the different percentages belong to similar concentrations). The dissolution in the duodenum is determined by the composition of the suspension (dissolved API and suspended solid particles) entering the gastric chamber. Due to the higher equilibrium solubility in pH 6.5 blank FaSSIF (~2.5 mg/mL), the transferred solid particles are expected to dissolve. A dose-proportional dissolution profile was observed in this compartment and the jejunum. Since most of the absorption takes place in the upper small intestine, linear pharmacokinetics may be assumed based on the dissolution results.

**Figure 5:**
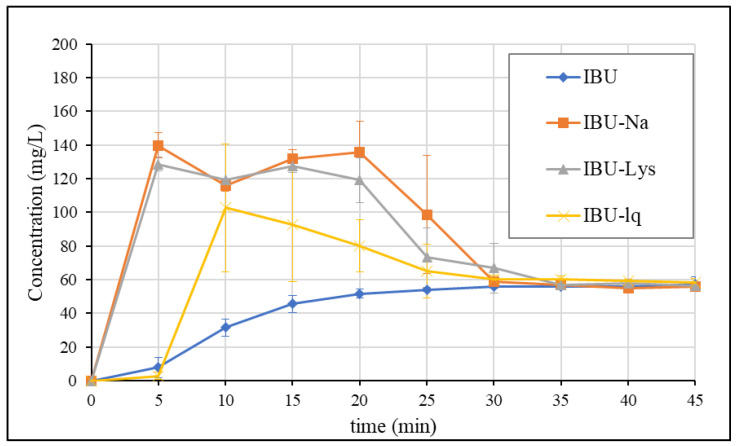
Gastric concentration profiles of ibuprofen formulations (1 × 200 mg) in blank biorelevant media.

The GIS dissolutions in blank biorelevant media were also performed with the other formulations. Figure 5 shows the obtained concentration profiles in the gastric compartment.

The results show that rapid-release products form supersaturated solutions that precipitate over time. By the end of the measurement, the concentration of the API approaches the equilibrium solubility (S_BlankFaSSGF_ = 56.3 mg/L) for all formulations. The degree of supersaturation is similar in the case of salt forms (IBU-Na: ~2.5× and IBU-Lys: ~2.3×) and somewhat less in the case of soft-gelatin capsules (IBU-lq: ~1.8×). The onset of release is delayed by ~5 min for IBU-lq, which is due to the disintegration of the capsule shell based on visual observation. The lack of supersaturation of IBU (free acid in conventional tablets) suggests that the obtained result of rapid-release products is a consequence of the applied formulation techniques (salt formation or pre-dissolved API).

**Figure 6:**
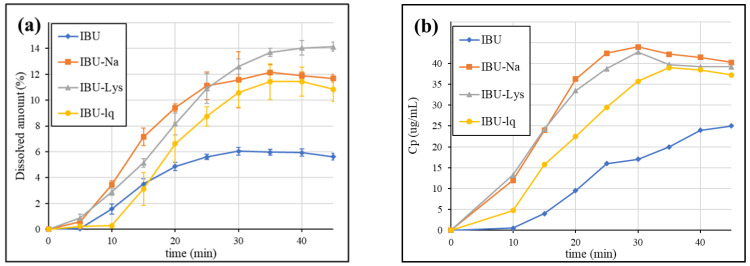
GIS duodenal dissolution in blank biorelevant media (**a**) vs. fasting BA study results (**b**) [23].

Figure 6 shows the dissolution curves in the duodenal chamber (a) compared to the first 45 min of the published clinical results (b) [23].

Based on the dissolution profiles of Figure 6a, the effect of gastric supersaturation results in a higher dissolved amount in the duodenum as well. All three rapid-release products reach a higher maximum concentration compared to the standard IBU formulation, which is consistent with the in vivo results. The plateau of IBU-Lys is slightly higher than that of IBU-Na and IBU-lq, which, however, was not experienced in vivo. It should be noted, though, that the formulations containing ibuprofen-lysinate salt tested in the in vitro and in vivo studies came from different manufacturers. In the case of IBU-lq, the delay in the onset of dissolution experienced in the gastric chamber persists in the duodenum and also appears in vivo.

**Figure 7:**
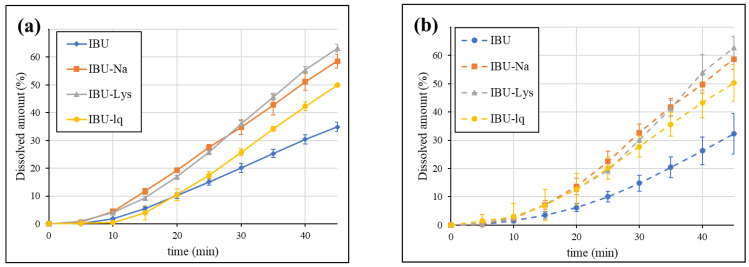
GIS sum of the amount dissolved in the duodenum and jejunum in blank biorelevant media (**a**) and biorelevant media (**b**).

The absorption of the API is expected in the entire upper small intestine, therefore, the sum of dissolution in the duodenum and jejunum compartments may correlate with the in vivo performance of the formulations. Thus, the results were also evaluated in this way. In addition to the GIS dissolutions in blank biorelevant media, the tests were also carried out in biorelevant media containing biomolecules. The obtained results are compared in Figure 7. The dissolution in the jejunum itself showed a very similar tendency to that of the sum of the two chambers. The results are shown in Figure S1 in the Supplementary Materials.

According to Figure 7, regardless of the addition of biomolecules, the dissolution rate of rapid-release formulations is higher than that of IBU (conventional tablet). The applied natural surfactants have only a small effect on the dissolution, which indicates that the increase in solubility (owing to the ionization caused by the pH shift between the stomach and the duodenum) is sufficient to dissolve the entering suspension. In general, the small intestinal dissolution of BCS Class IIa drugs in fasted conditions is expected to be much more influenced by the pH change (i.e., ionization) than by the presence of biomolecules (solubilizing effect). These findings are in agreement with previous study results [40]. The simplification of biorelevant buffers by omitting the addition of biomolecules may be a cost-effective way of the GIS analysis without affecting the predictivity of the method.

Overall, the GIS results highlighted a complex process leading to enhanced absorption of rapid-dissolving ibuprofen formulations: The initial supersaturation is followed by precipitation in the acidic stomach; however, the continuous emptying of the supersaturated suspension resulted in a higher dissolution rate at the higher pH of the duodenum and the jejunum. The multi-compartmental design, as well as the appropriate modelling of the gastrointestinal pH conditions and fluid volumes, were essential to achieve the desired predictivity. In contrast, the USP dissolution method using a high volume of pH 7.2 phosphate buffer to ensure sink condition was unable to differentiate between conventional and enhanced bioavailability formulations.

### 3.4. Establishment of the IVIVC Model

IVIVC is a predictive mathematical model describing the relationship between an in vitro property and a relevant in vivo response. Level A correlation, which represents a point-to-point relationship, is considered to be the most informative and is recommended by the authorities whenever possible [41]. The IVIVC model was established to justify the predictivity of the GIS method. The correlation was built on the in vitro and in vivo data of IBU (internal batch), and then the plasma profiles of the other formulations were simulated based on the dissolution results (external batches).

#### 3.4.1. Analysis of In Vivo Data

**Figure 8:**
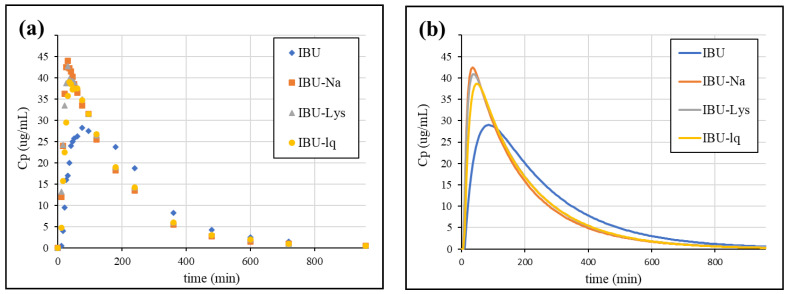
Published plasma concentration profiles (**a**) [23] vs. fitted curves (**b**).

The mean plasma concentrations of the fasting crossover pharmacokinetic study published by Legg et al. [23]. were first digitized and then fitted using the pK module (Model 14: two-compartmental pK model) of the WinNonLin IVIVC Toolkit. The in vivo profiles are presented in Figure 8.

**Figure 9:**
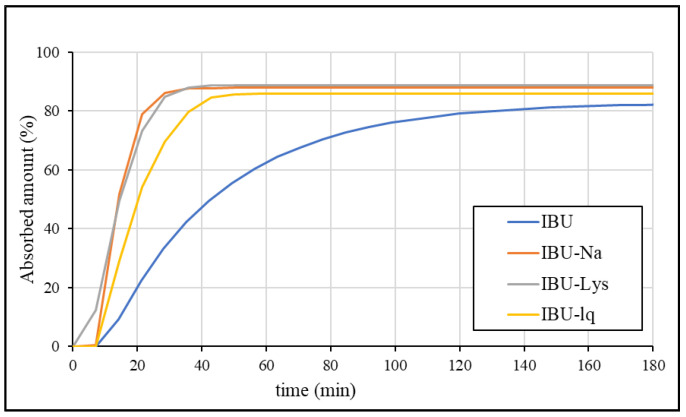
Absorption profiles obtained by deconvolution of clinical data [23].

The estimated parameters of the model describing the plasma profile of IBU were applied as input parameters to calculate the UIR function. The estimated parameters were A1 = 50.32, A2 = 0.007514, alpha1 = 0.004773, and alpha2 = 0.004006. The UIR function enabled the deconvolution of plasma concentration profiles, resulting in the fraction absorbed curves, which are shown in Figure 9.

#### 3.4.2. Fitting of In Vitro Dissolution Data

The sum of the amount of dissolved curves in the duodenum and jejunum chambers of the GIS using blank biorelevant media was fitted with the Weibull function. The estimated parameters of the functions are listed in Table 5.

**Figure 10:**
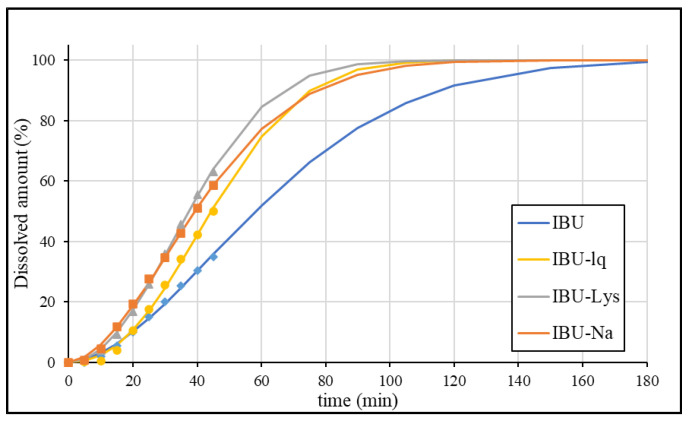
Fitted dissolution data using the Weibull function.

The calculated dissolution profiles fitted to the average data are shown in Figure 10.

Based on Figure 10, the calculated curves fit the experimental data well, however, extrapolation is required to describe the whole dissolution profile. Therefore, this phase of the profiles has a greater uncertainty. Comparing the in vitro (Figure 10) and the in vivo (Figure 9) data, it appears that there is a slightly greater difference in the absorption rate between IBU and the rapid-release formulations than in the observed dissolution rate.

#### 3.4.3. Correlation

**Figure 11:**
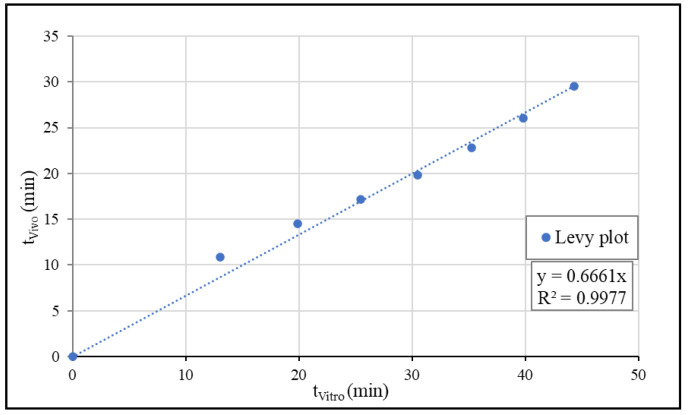
The Levy plot of IBU.

The in vitro dissolution (from Weibull fitting) and the in vivo absorption (from deconvolution) of IBU were correlated using the Levy plot. The times corresponding to nominally the same dissolution (t_Vitro_) and absorption (t_Vivo_) were plotted, and the relationship was estimated using linear regression. The Levy plot is presented in Figure 11.

#### 3.4.4. Simulation of Plasma Concentration Profiles

**Figure 12:**
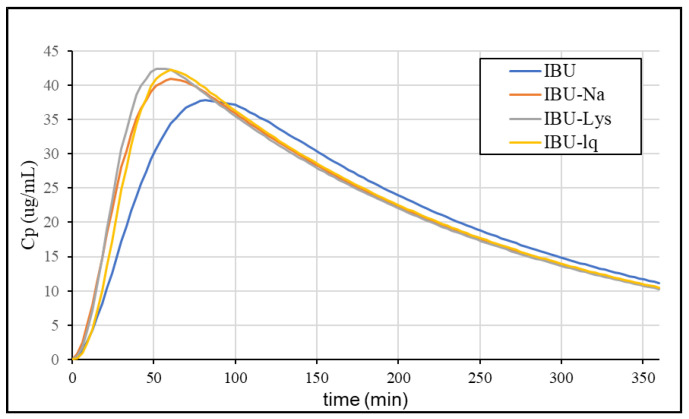
The simulated plasma concentration profiles of ibuprofen formulations.

Based on the estimated correlation, we calculated the absorption curves from the dissolution of the formulations and then convolved using the UIR function, which resulted in the plasma concentration profiles. The simulated profiles are shown in Figure 12.

The Cmax values and their ratio compared to IBU from the statistical analysis of the individual plasma concentration profiles, the mean curves, and the IVIVC prediction is summarized in Table 6.

According to the summarized Cmax values, the ratios predicted based on IVIVC correlate more with the statistical analysis of the individual profiles than with the ratio of the mean profiles. The statistical output of a clinical study provides the most relevant description of the differences between the formulations of interest, however, mean profiles are usually used for modelling purposes in the absence of published individual data. The simulation of the plasma concentration profiles using the established IVIVC model was able to predict the enhanced absorption rate of the rapid-dissolving ibuprofen formulations. Higher Cmax and lower tmax values were obtained compared to IBU. For both parameters, the differences were slightly underestimated. The rapid-release formulations were found to be similar to each other, which is also consistent with the in vivo data.

## 4. Conclusions

The advanced in vivo predictivity of the GIS system for BCS Class IIb and Class IIc compounds has previously been studied in the literature. In the present paper, the better in vivo predictivity of the method was also demonstrated for immediate release formulations containing BCS Class IIa compounds, to which less attention was paid before. The key factors resulting in the superiority of the GIS compared to the USP method were the multi-compartmental design, the biorelevant fluid volumes and the pH change, which enabled the modelling of the complex mechanism behind the advanced absorption of rapid-dissolving ibuprofen formulations. It was found that pre-dissolving or salt formation of poorly soluble acidic compounds leads to temporary supersaturation in an acidic medium, which, thanks to the continuous gastric emptying, affects the resulting concentration in the upper small intestine as well. Both dissolution and solubility results indicated that the role of gastrointestinal pH conditions in the in vivo dissolution of poorly soluble, acidic drug substances is more significant compared to the solubilizing effect of biomolecules. In conclusion, the multi-compartmental GIS model using blank biorelevant media was found efficient in predicting the in vivo performance of ibuprofen formulations. Predicting the in vivo behaviour and providing a better understanding of the absorption process can both contribute to the successful development of enhanced bioavailability formulations containing BCS Class IIa drugs.

## Figures and Tables

**Table 1:** Dosage form and active ingredients of the tested products.

Product	Manufacturer	API Form	Abbreviation
Advil 200 mg coated tablets	Pfizer Consumer Healthcare, Madison, NJ, USA	ibuprofen free acid	IBU
Advil 256 mg film-coated tablets	Pfizer Consumer Healthcare, Madison, NJ, USA	ibuprofen sodium	IBU-Na
Dolowill RAPID 342 mg film-coated tablets	Goodwill Pharma, Szeged, Hungary	ibuprofen lysinate	IBU-Lys
Advil ULTRA 200 mg soft-gelatin capsules	Pfizer Consumer Healthcare, Madison, NJ, USA	Ibuprofen in solution	IBU-lq

**Table 2:** Preparation of 1 L buffer solutions for GIS dissolution.

	Blank Biorelevant Media			Biorelevant Media		
	Blank FaSSGF	Blank FaSSIF	Blank FaSSIF conc.	Full FaSSGF	Full FaSSIF	Full FaSSIF conc.
NaCl	2.00 g	6.19 g	40.24 g	2.00 g	6.19 g	40.24 g
NaOH	-	0.40 g	2.60 g	-	0.40 g	2.60 g
NaH_2_PO_4_. H_2_O	-	3.96 g	25.74 g	-	3.96 g	25.74 g
SIF powder	-	-	-	0.06 g	2.25 g	14.63 g
Pepsin	-	-	-	0.10 g	-	-
pH adjustment	cc. HCl:purified water = 1:1	1M NaOH	-	cc. HCl:purified water = 1:1	1M NaOH	-

**Table 3:** The pH-dependent equilibrium solubility of ibuprofen at 37 °C.

pH	S_pH_ ± SD (µg/mL) ^1^	logS_pH_ (mol/L)
1.92	70.8 ± 3.0	−3.46
3.96	124 ± 13	−3.22
5.95	1910 ± 70	−2.03
7.17	32,033 ± 4135	−0.81
8.02	300,000 ± 6500	0.16
14	734,000 ± 30,500	0.55

^1^ the results at each pH are the mean of 3 parallel measurements.

**Table 4:** Equilibrium solubility of ibuprofen in biorelevant media at 37 °C.

Solvent	S_pH_ ± SD (µg/mL) ^1^
FaSSGF blank, pH 1.6	56.3 ± 0.6
FaSSGF, pH 1.6	56.0 ± 0.5
FeSSGF-acetate, pH 4.5	194 ± 2
FeSSIF blank, pH 5.0	416 ± 12
FeSSIF, pH 5.0	2103 ± 56
FaSSIF blank, pH 6.5	2513 ± 15
FaSSIF, pH 6.5	3160 ± 31

^1^ the results at each pH are the mean of 3 parallel measurements.

**Table 5:** Estimated parameters of the Weibull functions.

Formulation	B	MDT (min)
IBU	1.759	71.55
IBU-Na	1.786	48.23
IBU-Lys	2.087	44.55
IBU-lq	2.274	52.18

**Table 6:** Observed [23] vs. predicted pharmacokinetic data of ibuprofen formulations.

	Clinical Data Statistical Analysis of Individual Profiles			Clinical Data Mean Plasma conc. Profiles		IVIVC Prediction from GIS Dissolution		
Formulation	Cmax	Ratio	tmax	Cmax	Ratio	Cmax	Ratio	tmax
IBU	37.70	N/A	82.1	28.25	N/A	37.80	N/A	81.7
IBU-Na	47.00	1.25	35.2	44.00	1.56	41.00	1.09	60.5
IBU-Lys	49.90	1.32	35.1	42.75	1.51	42.40	1.12	61.4
IBU-lq	46.80	1.24	40.0	39.00	1.38	42.20	1.12	60.5

## Data Availability

Not applicable.

## Supplementary Materials

The following supporting information can be downloaded at: https://www.mdpi.com/article/10.3390/pharmaceutics15030753/s1, Figure S1: GIS jejunal dissolution in blank biorelevant media.

## Abbreviations

API	Active pharmaceutical ingredient
ASD	Artificial stomach duodenal model
BCS	Biopharmaceutics Classification System
BR	Britton–Robinson
DGM	Dynamic Gastric Model
GIS	Gastrointestinal Simulator
HPLC	High-Performance Liquid Chromatography
HGM	Human Gastric Simulator
IVIVC	In vitro–In vivo correlation
NSAID	Non-Steroidal Anti-Inflammatory Drug
S_pH_	PH-dependent solubility
TIM	TNO gastro-Intestinal Model
UIR	Unit impulse response
USP	United States Pharmacopoeia
