# Supplementary figures and images for: Multi-Compartmental Dissolution Method, an Efficient Tool for the Development of Enhanced Bioavailability Formulations Containing Poorly Soluble Acidic Drugs
Source: Pharmaceutics. 2023 Feb 24;15(3):753. doi: 10.3390/pharmaceutics15030753 (PMC10051608; doi:10.3390/pharmaceutics15030753)

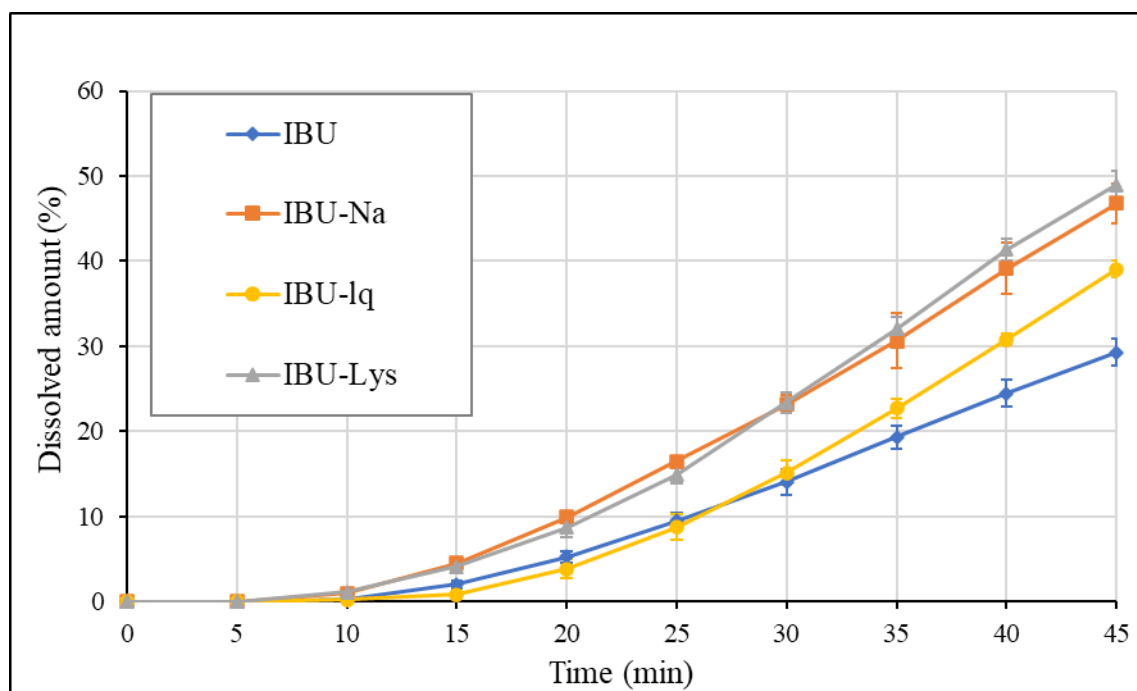

**Figure S1** GIS jejunal dissolution in blank biorelevant media

Supplement: Supplementary file 1 [file pharmaceutics-15-00753-s001.zip › pharmaceutics-2197449-supplementary.pdf]
